# Comparing phenotypic manifolds with Kompot: Detecting differential abundance and gene expression at single-cell resolution

**DOI:** 10.1101/2025.06.03.657769

**Published:** 2025-06-07

**Authors:** Dominik J. Otto, Erica Arriaga-Gomez, Elana Thieme, Ruijin Yang, Stanley C. Lee, Manu Setty

**Affiliations:** 1 Basic Sciences Division, Fred Hutchinson Cancer Center, Seattle WA; 2 Computational Biology Program, Public Health Sciences Division, Seattle WA; 3 Translational Data Science IRC, Fred Hutchinson Cancer Center, Seattle WA; 4 Translational Science and Therapeutics Division, Fred Hutchinson Cancer Center, Seattle WA; 5 Molecular and Cellular Biology Program, University of Washington, Seattle WA

## Abstract

Kompot is a statistical framework for holistic comparison of multi-condition single-cell datasets, supporting both differential abundance and differential expression. Differential abundance captures changes in how cells populate the phenotypic manifold across conditions, while differential expression identifies condition-specific changes in gene regulation that may be localized to particular regions of that manifold. Kompot models the distribution of cells and gene expression as continuous functions over a low-dimensional representation of cell states, enabling single-cell resolution inference with calibrated uncertainty estimates. Applying Kompot to aging murine bone marrow, we identified a continuum of shifts in hematopoietic stem cell and mature cell states, transcriptional remodeling of monocytes independent of compositional changes, and divergent regulation of oxidative stress response genes across cell types. By capturing both global and cell-state–specific effects of perturbation, Kompot reveals how aging reshapes cellular identity and regulatory programs across the hematopoietic landscape. This framework is broadly applicable to dissecting condition-specific effects in complex single-cell landscapes.

## Introduction

Multi-condition single-cell datasets offer new opportunities to dissect how mutations or other perturbations affect both differentiation trajectories and disease phenotypes^1–4^. Systematic analyses of such datasets require characterization of two key aspects of condition-induced variability: differential abundance and differential expression.

**Figure 1:**
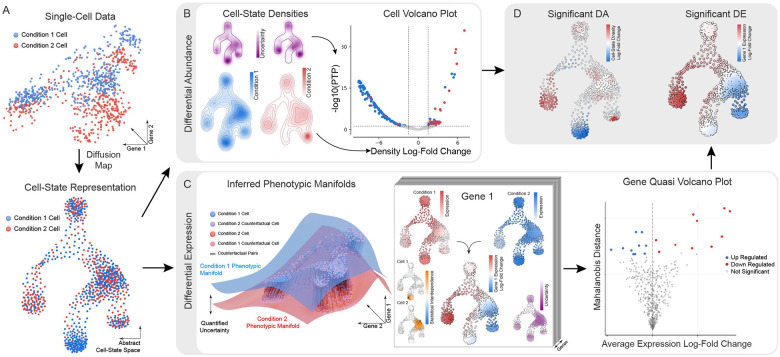
Overview of Kompot’s framework for differential abundance and expression analysis. (A) Illustration of high-dimensional single-cell transcriptomic data from two conditions, projected into a shared low-dimensional cell-state space using diffusion maps. (B) Differential abundance analysis in Kompot. Condition-specific cell-state densities with associated uncertainty estimates are computed and compared to yield density log-fold changes. Significance is quantified using posterior tail probability (PTP), visualized in a quasi-volcano plot. (C) Differential expression analysis. Left: schematic of inferred phenotypic manifolds for both conditions with counterfactual cell pairs illustrating expression comparison. Middle: gene-specific differences are illustrated for one example gene, showing condition-specific expression levels, statistical interdependence, and uncertainty. Right: A quasi-volcano plot summarizes gene-level results with the average log-fold change on the x-axis and Mahalanobis distance (accounting for variance and covariance) as a multivariate significance score on the y-axis. (D) Visualization of differential abundance (left) and differential expression (right) across the cell-state space, colored by log-fold change magnitude.

Differential abundance refers to differences in the composition of cell states between two conditions (Fig. 1A, B). Even in the absence of overt phenotypic shifts such as loss or gain of broad cell types, changes in the frequency of specific cell states can provide early indicators of disease^5, 6^ and response to treatment^7, 8^. These changes are often nuanced and cell-type specific, necessitating statistical approaches that rigorously assess significance while accounting for underlying biological variability.

Perturbations can also reshape the phenotypic landscape itself through differential expression, where gene expression changes that would be unlikely or impossible in unperturbed conditions can emerge^9^ (Fig. 1A, C). These transcriptional responses reveal condition-specific molecular programs and regulatory networks that often manifest as subtle yet systematic changes across multiple states^9^ and can frequently be masked by the inherent variability within biological systems.

Several computational approaches have been developed for comparative analysis of multi-condition single-cell data with differential abundance receiving greater attention^10–15^. A number of these methods rely on discretizations such as clustering cells into discrete groups to stabilize variability estimates. While facilitating interpretation, such discretizations lead to loss in resolution when changes are gradual or occur along continuous trajectories (Supp. Fig. 1A). Additionally, many existing methods lack principled significance measures, making it difficult to distinguish genuine biological signals from technical noise.

Here, we introduce Kompot, a novel computational framework that provides a continuous and statistically rigorous approach for differential abundance and differential expression testing using multi-condition single-cell data. Kompot leverages Gaussian Process (GP) modeling to estimate continuous functions across the cell-state space. This approach enables quantification of differential abundance and differential expression at single-cell resolution, while incorporating uncertainty estimates to provide statistically calibrated comparisons. By addressing both aspects of multi-condition analysis within a unified statistical framework, Kompot provides a comprehensive toolkit for extracting biological insights from increasingly complex single-cell datasets across a range of biological systems.

## Results

### Overview of the Kompot modeling framework

A single-cell RNA-seq dataset provides a snapshot of cellular states in a biological system, with the molecules measured representing a sample of the transcriptome of the cell. To characterize the system and compare it to similar systems under different conditions (such as mutations or other perturbations), we need to understand what this data tells us about the true composition of transcribed genes in a cell and the true distribution of cell states in the system. With Kompot we leverage high-dimensional, continuous cell-state representations and Bayesian Inference to learn both, the composition of the system and transcriptomes of each cell, enabling a holistic comparison of biological systems at single-cell resolution with uncertainty quantification. This comparison is divided into differential cell-state abundance that reflect changes in cell-state composition, and differential-expression that reflect changes in the transcriptome of cells in equivalent states.

The input to Kompot is a latent representation of co-embedded multi-condition single-cell data. This latent representation can incorporate batch effect correction^16, 17^ to ensure that cell states considered equivalent share similar locations in that state space (Supp. Fig. 1B). From this foundation, Kompot employs diffusion maps^18^ to derive a cell-state representation with biologically meaningful structure (Fig. 1A). Diffusion maps are particularly effective for single-cell analysis as they capture the intrinsic geometry of the data while providing biologically meaningful measures of cell-to-cell distance^19,20^. This distance metric supports both accurate local abundance estimation and principled information sharing when mapping from cell-state space to gene expression, enabling the statistical comparisons at the core of Kompot’s differential testing framework.

### Kompot differential abundance testing

To quantify differences in cell-state composition between conditions, Kompot first constructs condition-specific density functions on the diffusion map representation. For each condition, Kompot computes a separate continuous density function (Fig. 1B, Supp. Fig. 2A–B) using our Mellon framework^21^. Mellon leverages nearest-neighbor distances within a Gaussian Process model to estimate continuous density functions that capture the distribution of cells across the state space^21^. The continuous nature of these density estimates enables evaluation at any position within the cell-state space, including states predominantly observed in the alternate condition (Supp. Fig. 2B). By computing the ratio of density estimates at corresponding positions in the cell-state space, Kompot derives a measure of differential abundance that quantifies the fold-change in cell density between conditions at single-cell resolution (Supp. Fig. 2C).

While the ratio of density estimates provides a measure of fold-change, rigorous comparison between conditions requires statistical assessment of significance. Kompot addresses this challenge by computing uncertainty estimates for each density function. These uncertainty estimates account for two critical factors: limited data availability in regions of the cell-state space and inherent uncertainty through the sparse function representation. By incorporating these uncertainty estimates into the statistical framework, Kompot calculates posterior tail probabilities (PTPs), an alternative for p-values, for differential abundance at each cell state. This enables the generation of volcano plots for differential abundance testing where each point represents a single cell-state (Fig. 1B), allowing for threshold-based significance assessments. Thus, Kompot can identify subtle cell-state transitions and rare subpopulations that would likely be undetected by clustering-based approaches, providing insights into the continuous spectrum of cellular responses to perturbations (Fig. 1D).

### Kompot differential expression testing

For differential expression testing between conditions, Kompot constructs condition-specific mappings from cell-states to gene expression space (Fig. 1A, C). These mappings are computed using Gaussian Processes (GPs)^22^, by predicting log-transformed gene expression as a continuous function of the diffusion map coordinates. The non-linear relationship between diffusion maps and gene expression is effectively captured by GPs^22^, essentially serving as a rigorous approach to gene expression imputation. The continuous nature of these mapping functions allows Kompot to estimate gene expression in one condition for cell states observed in another, producing counterfactual expression profiles that reflect how those same cell states would behave under different conditions (Fig. 1C). By computing the ratio between the actual expression in a cell and its counterfactual expression, Kompot quantifies gene expression-fold changes for every gene at single-cell resolution across the entire phenotypic landscape (Fig. 1C).

Given the large number of genes evaluated, we require a robust significance measure that allows us to identify the most relevant genes, accounting for both uncertainty in the gene expression function and statistical interdependence of imputed values. Kompot therefore employs the Mahalanobis distance, which measures the distance of a point from a multivariate normal distribution, considering both variance and covariance^23^. It effectively measures how many standard deviations a gene expression shift is away from expected variation, while accounting for the correlation structure between neighboring cell states, making it an ideal significance measure for differential expression. Kompot therefore uses Mahalanobis distances as the score in a quasi-volcano plot (Fig. 1C) to identify significantly differentially expressed genes either in the full population of cells or in specific subsets (Fig. 1D).

### Differential Abundance in Aging Bone Marrow

**Figure 2:**
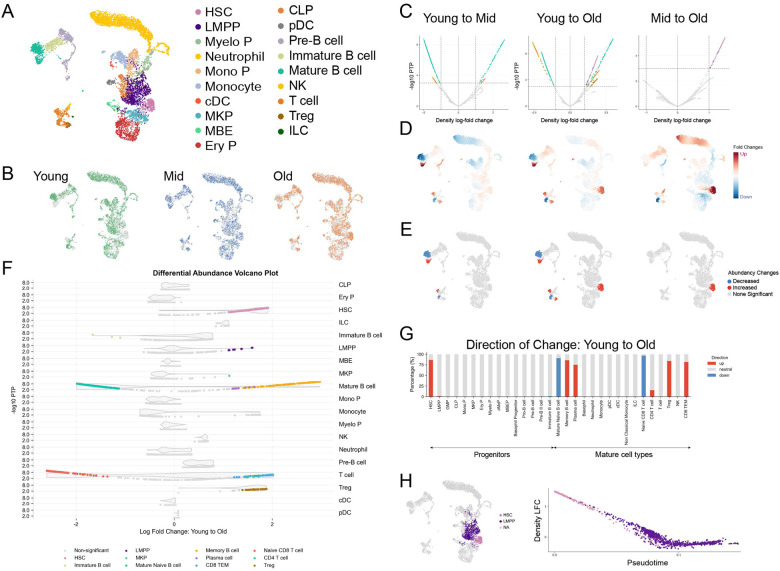
Kompot differential abundance analysis in aging hematopoiesis. (A) UMAP projection of aging murine bone marrow scRNA-seq dataset, colored by medium-resolution cell types. (B) UMAPs highlighting cells from mice grouped by age: Young (10 weeks), Mid (63 weeks), and Old (103 weeks). (C) Differential abundance results for the Young to Mid (left), Young to Old (middle) and Mid to Old (right) comparisons. Quasi-cell volcano plot with density log-fold change on the x-axis and Posterior Tail Probabilities (PTP) on the y-axis. Significantly different cell-states (absolute log fold change > 1, PTP < 10^−3^) are highlighted by their cell type annotations from (A). (D) UMAPs displaying the estimated local density log-fold changes across the full joint cell-state space derived from all samples (Young, Mid, and Old) for comparisons in C. (E) UMAPs highlighting the subset of cell states with statistically significant abundance changes, as defined by the thresholds shown in the volcano plots in C. (F) Differential abundance volcano plots showing density log-fold changes across cells, stratified by major cell type and colored by subtype in the Young to Old comparison. y-axis indicates posterior tail probability. Violin plots in the background indicate abundance of cells at the respective density log-fold change in this group. (G) Direction and magnitude of abundance changes per cell type in the Young to Old comparison. Bars show the fraction of significantly changing cell-states within each annotated cell type, color-coded by the direction of change. Cell types are ordered along a progenitor-to-mature axis. (H) Kompot results for early hematopoietic progenitors. Left: UMAP highlighting HSC, and LMPP subsets. Right: Scatterplot of cells colored by density log-fold change along pseudotime (x-axis), showing dynamic abundance shifts during early differentiation.

Aging is a compelling biological context for evaluating differential abundance methods, as it induces progressive and often subtle shifts in cellular composition rather than dramatic phenotypic transformations. Hematopoiesis has emerged as a particularly informative system for studying aging^24^, characterized by declining stem cell function^25^, reduced lymphocyte production^26^, and increased susceptibility to myeloid malignancies^27^. To investigate these age-related changes, we generated a novel scRNA-seq dataset of murine bone marrow at 10, 63, and 103 weeks of age, representing young, middle-aged, and older timepoints (Supp. Fig. 3A). After quality control verification (Supp. Fig. 3B, C), we successfully identified most hematopoietic cell types across all ages (Fig. 2A, B, Supp. Fig. 4), providing us with a rich dataset to characterize cell-state changes and gene expression changes in aging hematopoiesis using Kompot.

We applied Kompot differential abundance testing on our aging hematopoiesis dataset to determine age-dependent composition changes (Fig. 2C–E). We first derived a unified cell-state representation for all cells using diffusion maps. We then calculated age-specific density functions using the subsets of cells from each timepoint (Supp. Fig. 5A–B). We evaluated these individual density functions at all cell-states in the dataset to derive condition-specific densities and associated uncertainties (Supp. Fig. 5C–D). This approach enabled pairwise comparisons that quantify age-related changes in cell-type composition (Fig. 2C–D). A key advantage of our framework is the ability to set thresholds based on both effect size and statistical significance at single-cell resolution, allowing for identification of cell states with significant differential abundance rather than relying solely on fold-change cutoffs (Fig. 2C–E).

Our analysis revealed significant expansions and contractions in distinct cell populations over time. Comparing young to old mice, we observed an expansion of the hematopoietic stem cell (HSC) compartment and a marked shift from naïve to memory cells in both B-cell and T-cell compartments (Fig. 2F–G). The depletion of naïve B-cells and an enrichment of memory B-cells in older mice is a well-characterized phenomenon of hematopoietic aging^26^. This shift reflects fundamental changes in differentiation dynamics: naive B-cells rely on a continuous influx from hematopoietic progenitors, whereas memory B-cells accumulate over time due to antigen exposure. The observed decline in naïve B-cells reflects the reduced lymphoid differentiation potential of aged HSCs^28^, while the expansion of memory B-cells, including age-associated B-cells, is driven by persistent immune activation^29^. Similarly, the T-cell compartment displayed a significant reduction in naïve T-cells with a concurrent increase in memory T-cells, a hallmark of immunosenescence that contributes to diminished adaptive immune responses in aging^30^.

An increased accumulation of hematopoietic stem cells (HSCs) is the most significant change we observed in the progenitor cells (Fig. 2F–G). Progressive enrichment of HSCs with age is a well-documented phenomenon^31^. HSCs reside at the top of the hematopoietic hierarchy, maintaining blood production through self-renewal and differentiation into mature immune and blood cells. With aging, HSC function declines, leading to accumulation in the bone marrow while their ability to differentiate into different lineages becomes impaired^31^. While the accumulation of aged HSCs has been previously observed, it has traditionally been quantified using bulk measurements or clustering-based approaches. We took advantage of the single-cell resolution of Kompot to better characterize the age-induced change in HSCs. By comparing the differential abundance fold-change along pseudotime ordering^19^, we observed that the expansion of HSC population is in fact a subtle shift in cell-state, rather than a global increase in a predefined population (Fig. 2H). Specifically, HSCs in older mice progressively shift towards a less differentiation-primed state, creating a continuum of altered stem cell states rather than a uniform expansion (Fig. 2H). This subtle but consequential repositioning along the differentiation trajectory provides a possible explanation for the functional decline in aged HSCs, revealing how changes in cell abundance are accompanied by fundamental alterations in cellular function and developmental potential.

Comparing differential abundance results across ages, we observed that changes in mature cell populations predominantly occurred between young and middle-aged mice (Fig. 2D, Supp. Fig. 6A), while alterations in the HSC compartment were more pronounced between middle-aged and older mice (Fig. 2E, Supp. Fig. 6B). This temporal separation suggests a sequential progression of aging, where compositional shifts in differentiated cells precede fundamental changes in stem cell states. The early alterations in mature immune cells may represent initial adaptive responses to aging, while the later HSC changes potentially indicate more persistent reprogramming of the hematopoietic system.

Our results demonstrate the ability of Kompot to identify subtle but biologically meaningful abundance changes between conditions. By combining a continuous representation of cell states with robust statistical measures, Kompot enables precise detection of population-level changes while maintaining single-cell resolution.

### Differential Gene Expression in Aging Hematopoiesis

**Figure 3:**
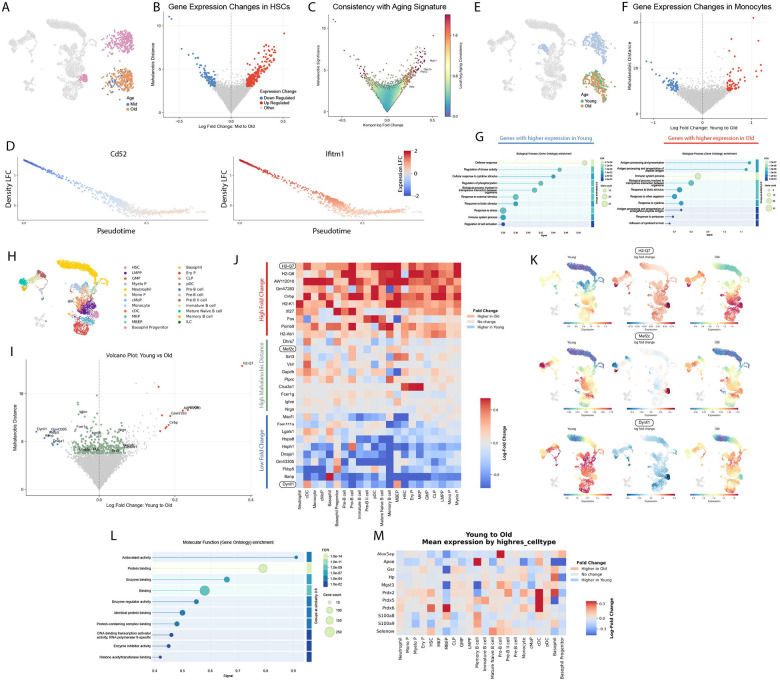
Kompot differential expression analysis in aging hematopoiesis. (A) UMAP from Fig. 2A, with HSCs highlighted. (B) Differential expression results for Mid to Old HSCs. Quasi-cell volcano plot with expression log-fold change on the x-axis and Mahalanobis distance on the y-axis. Significantly different genes (absolute log fold change > 0.15, Mahalanobis distance > 3) are highlighted. (C) like (B) colored by weighted average of reported “Aging Consistency” using standard Gaussian Kernel after scaling x- and y-axis to standard deviation 1. (D) Same plot as Fig. 2H, colored by Mid to Old HSC gene expression log fold change for a gene downregulated with age (Cd52, left) and an upregulated gene (Ifitm1, right). (E) UMAP from Fig. 2A, with monocytes highlighted. (F) Same as (B), for Young to Old monocytes. (G) Gene ontology analysis from STRING^37^ for downregulated genes (left) and upregulated genes (right) from Fig. 2F. (H) UMAP for Fig. 2A, highlighting all cell types except T and NK cells (I) Differential expression results for all cells in Fig. 2H for Young to Old comparisons. Top 10 genes with positive fold change and high Mahalanobis distance are shown in red, bottom 10 genes with negative fold change and high Mahalanobis distance are shown in blue, and genes with log absolute fold change but high Mahalanobis distance are shown in green (absolute log fold change < 0.07, Mahalanobis distance > 6). 20 genes are labeled. (J) Heatmap showing Young to Old gene expression log fold changes across different cell types for genes highlighted in (I). (K) Top: Imputed expression of gene H2-Q7 using young cells (left), using old cells (right) and fold change from young to old (middle). Middle, Bottom: Same as the top row for Mef2c and Dynll1. These genes serve as examples of genes with high positive fold change, high Mahalanobis but low absolute fold change, and high negative fold change respectively. (L) Gene ontology analysis using genes with high Mahalanobis distance and low absolute fold change from (I). (M) Heatmap showing Young to Old gene expression log fold changes across different cell types for genes involved in antioxidant activity.

We applied Kompot differential expression testing on our mouse aging hematopoiesis dataset to characterize age-related transcriptional changes (Fig. 3). We first inferred age-specific mapping functions from diffusion map coordinates to log-normalized gene expression values, using the subset of cells from each timepoint. We then used these mappings to impute gene expression and associated uncertainty at all cell-states across the dataset. This enabled us to compute gene-level fold changes by comparing observed expression values to their counterfactual predictions under alternative age conditions. To assess significance, we calculated Mahalanobis distances for each gene across specified groups of cells, quantifying deviations from expected variation while accounting for the covariate structure among neighboring cell states. We excluded T and NK cells for this analysis since do not differentiate in the bone marrow (Fig. 3H).

We first applied our approach to characterize gene expression changes in HSCs, a population known to undergo functional and transcriptional changes with age^31^, by comparing HSCs from middle-aged to old-aged mice (Fig. 3A–B). Aging in HSCs has been extensively studied, and the Hematopoietic Aging Atlas has synthesized results from multiple datasets to identify genes consistently up- or downregulated with age^32^. For each gene, this atlas assigns a consistency score that reflects the reproducibility of age-associated expression changes across studies^32^. Kompot’s differential expression results strongly prioritized genes with the highest consistency scores in the Hematopoietic Aging Atlas, with genes showing large fold changes and Mahalanobis distances highly enriched for reproducible aging signatures (Fig. 3C, Supp. Fig. 7).

Our differential abundance analysis revealed that HSCs in older mice shift towards a less differentiated state along the cell-state continuum (Fig. 2H). We next asked whether the genes differentially expressed in aged HSCs reflected this shift. Indeed, we found that gene expression fold changes were structured along the same continuum: genes exhibited larger fold changes in cell states that were more differentially abundant (Fig. 3D). These results highlight the transcriptional changes in HSCs that accompany and may underlie the shift toward a less differentiated state with age,

We next investigated age-related gene expression changes in mature cell types. We focused on changes from young to old mice since the abundance changes in mature cell types were most pronounced in this comparison (Fig. 2D–F). To distinguish expression changes from abundance-driven effects, we restricted our analysis to cell types that did not show *significant compositional changes*. Monocytes exhibited clear transcriptional changes despite stable abundance (Fig. 3E–F). Gene ontology analysis revealed that downregulated genes were enriched in core monocyte/macrophage functions such as defense response (Fig. 3G), consistent with their reduced functional capacity with age^33^. In contrast, upregulated genes were enriched for antigen processing and presentation pathways, driven by increased expression of MHC class II genes (Fig. 3G), which have been implicated in age-related immune remodeling^34^. Notably, our results recapitulate findings from a previous study that identified H2-Aa, H2-Ab1, H2-Eb1, Cd74, and Aw112010 as the top dysregulated genes in aging monocytes across both mice and humans, with only Igkc surpassing H2-Eb1 in our ranking^34^. The near-perfect agreement with experimentally validated gene sets underscores the power of our differential expression testing approach to detect biologically meaningful transcriptional shifts. Gene expression differences in other mature cell types were not as pronounced but did show a tendency for upregulation of MHC Class II genes similar to monocytes (Supp. Fig. 8).

Our use of Mahalanobis distance as a significance measure enables Kompot to identify genes that exhibit coordinated but opposing changes across subpopulations. For example, consider a gene that is upregulated in one cell type and downregulated in another. While the net fold change may be close to zero, the Mahalanobis distance remains high, reflecting a statistically significant deviation from expected variation if unaffected. This occurs because such a pattern is unlikely under the local covariance structure of the cell-state space, where neighboring states are typically expected to exhibit similar expression patterns. As a result, Kompot can detect structured differential expression that would be missed by conventional fold-change-based approaches.

To test this capability, we applied Kompot differential expression testing between young and old cells across all cell-types that differentiate in the bone marrow (Fig. 3H–I). The top genes with both high positive fold change and high Mahalanobis distance were strongly enriched for MHC class II genes, consistent with our monocyte-specific results and indicating that MHC class II upregulation is a general feature of hematopoietic aging (Fig. 3J). Likewise, genes with high Mahalanobis distance and strong negative fold change were consistently downregulated across multiple cell types (Fig. 3J). In contrast, genes ranked highly by Mahalanobis distance but with low absolute fold change exhibited divergent expression patterns across cell types, with upregulation in some and downregulation in others (Fig. 3J). To illustrate these distinctions, we visualized expression levels of representative genes from each category across the cell-state manifold (Fig. 3K). Genes selected by fold change showed broad, monotonic shifts in expression, whereas Mahalanobis-selected genes revealed distinct, cell-type-specific regulation (Fig. 3K).

We next performed gene ontology analysis to assess the biological relevance of genes with high Mahalanobis distance but low absolute fold change. Antioxidant activity emerged as the most significantly enriched term (Fig. 3L), a pathway known to be impacted by aging^35^. Notably, our results indicate that while antioxidant activity is broadly impacted across hematopoietic cell types, the direction of expression changes of the pathway genes varies across genes and cell types, suggesting a context-specific adaptation to aging (Fig. 3M). Oxidative stress is a known driver of HSC dysfunction, promoting DNA damage, apoptosis, and biased differentiation toward myeloid lineages^35^. The differential expression of antioxidant pathway genes observed in our study likely represents a compensatory mechanism to mitigate reactive oxygen species (ROS) accumulation in aged HSCs^35^.

These results demonstrate that Mahalanobis distance captures biologically meaningful heterogeneity in gene expression and provides a powerful approach for aggregating structured expression differences across cell states without requiring prior clustering, making it especially suited for complex, continuous single-cell landscapes.

## Discussion

Kompot provides a flexible statistical framework for quantifying condition-specific effects in single-cell data, modeling both cell abundance and gene expression as continuous functions over the cell-state manifold. By estimating condition-specific densities and expression profiles using Gaussian processes, Kompot performs differential analysis at single-cell resolution while accounting for uncertainty and spatial structure in the data. This approach supports biologically grounded interpretations of perturbation effects, whether they manifest as global shifts in population structure, localized transcriptional responses, or context-specific coordination between abundance and expression changes.

Applying Kompot to aging murine bone marrow revealed both well-established and previously unrecognized features of hematopoietic aging. Kompot recapitulated known expansions in memory lymphocytes and age-associated B cells, while resolving more subtle shifts in hematopoietic stem cell states that do not correspond to discrete population changes. In the transcriptomic landscape, Kompot uncovered gene expression programs that are either conserved or cell-type specific, including divergent regulation of oxidative stress pathways. These findings demonstrate how modeling continuous phenotypic structure supports more nuanced detection of biological change. Beyond aging, Kompot is broadly applicable to studies of development, disease progression, and response to perturbation, where condition-induced effects often unfold along continuous, high-dimensional trajectories.

## Methods

### Kompot framework

Kompot is a tool for comparing single-cell phenotypic manifolds across conditions. It provides frameworks for both differential abundance testing and differential gene expression testing at single-cell resolution using multi-condition datasets. Differential abundance testing enables the detection of abundance shifts of cell-states across the phenotypic manifold, without requiring predefined cell-type boundaries. This is achieved by estimating condition-specific cell-state densities using the Bayesian inference scheme implemented in Mellon. In parallel, through differential gene expression testing, Kompot identifies gene expression changes across the same manifold, capturing subtle but structured transcriptional shifts that may vary across cell states and may not follow consistent directionality To do so, Kompot leverages the Gaussian process inference implemented in Mellon to estimate condition-specific gene expression functions for thousands of genes within minutes, while explicitly sharing information across neighboring cell states to address sparsity and enable statistically meaningful comparisons

### Differential Abundance

This section explains the methods of Kompot’s differential abundance (DA) analysis framework. Our density quantification relies on the Gaussian Process (**GP**) estimation implemented in Mellon. This estimation process is a Bayesian inference that provides us with both a “best estimate” function that maps each point in the cell-state space to a density value $\mu \left(x_{i}\right)$, and a quantification of uncertainty $\sigma \left(x_{i}\right)$. Since we infer these quantities independently for each condition, the posterior distribution for the density change from condition a to condition b at any cell state $x_{i}$ is given by

$$\delta \left(x_{i}\right)\sim 𝒩\left(\mu_{b}\left(x_{i}\right)-\mu_{a}\left(x_{i}\right),\sigma_{a}^{2}\left(x_{i}\right)+\sigma_{b}^{2}\left(x_{i}\right)\right).$$

Therefore, our density log-fold change is defined by $\mu_{b}\left(x_{i}\right)-\mu_{a}\left(x_{i}\right)$, and our uncertainty quantification by $\sigma_{a}^{2}\left(x_{i}\right)+\sigma_{b}^{2}\left(x_{i}\right)$. Notably, using the correct intrinsic dimensionality, Mellon reports density in the unit log number of cells per volume.

To make density values comparable across samples with different cell counts, we can transform this unit into log fraction of cells per volume by subtracting the log total number of cells. Therefore, we normalize density in Kompot using

$$\mu_{a}^{\prime }\left(x_{i}\right)=\mu_{a}\left(x_{i}\right)-\text{log}N_{a}$$

where $N_{a}$ represents the total number of cells measured for condition a. For this normalization to be valid, it is essential, that we assume the correct intrinsic dimensionality of the phenotypic manifold. To that end we use Mellon dimensionality estimation that is assumed to be constant across the dataset.

Finally, we can define the Kompot’s local density log-fold change statistic as

Δxi=μbxi−μaxi−logNaNb.

Additionally, we provide a p-value-like significance value through the posterior tail probability (**PTP**). For example, if the density change $\Delta \left(x_{i}\right)$ is positive, then we compute $P\left(\delta \left(x_{i}\right)<0\right)$, and $P\left(\delta \left(x_{i}\right)>0\right)$ when $\Delta \left(x_{i}\right)$ is negative. The PTP quantifies the probability that the true fold change goes into the opposite direction of our best estimate, given our data and modeling assumptions. Since our posterior distribution is a normal distribution, the local Kompot PTP is given by:

$$PTP\left(x_{i}\right)=\Phi \left(\frac{-\left|\Delta \left(x_{i}\right)\right|}{\sqrt{\sigma_{a}^{2}\left(x_{i}\right)+\sigma_{b}^{2}\left(x_{i}\right)}}\right)$$

were Φ is the cumulative distribution function of the standard normal distribution.

### Differential Expression

Similar to the density differences, we also leverage Mellon’s GP implementation (Supp. Note 1) for differential expression (DE) analysis. In contrast to the density inference algorithm, this DE method directly conditions the gene-expression functions on the measured data, as classically done in GPs. For each condition, the goal is to compute a gene-expression function that predicts an expression value for each gene at any location in the cell-state space, specific to that condition. These functions provide a smooth and continuous representation of gene expression across the manifold and allow for quantifying differential expression between conditions at any point in state space, effectively integrating all data points without requiring predefined cluster boundaries.

The resulting gene-expression functions and uncertainty estimation can be used equivalently to the cell-state density functions to compute local gene-expression differences, featuring local fold change and PTP. However, apart from the question in which cell-state a gene may be differentially expressed, we want to answer if a gene asserts any significant DE within a cell population or across the entire cell-state space, effectively aggregating measurements across multiple cells.

This comes with an important caveat: The GP based gene-expression functions are an imputation that already accumulates measurements of multiple cells, and the resulting gene-expression values $f\left(x_{i}\right)$ at different cell states $x_{i}$ are not independent measurements that can be aggregated directly. This caveat can be addressed very directly through Mellon’s uncertainty quantification: It covers not only the variance for a function value $f\left(x_{i}\right)$ but also the covariance to other function values $f\left(x_{j}\right)$ based on the similarity of $x_{i}$ and $x_{j}$. This covariance can be understood as a statistical connection between the expressions of two cell states that comes from our assumption that genes change expression smoothly throughout cell-state space. Formally, under our modelling assumption, the true gene expression values f(x) at cell states $x={\left\{x_{i}\right\}}_{i=1}^{m}$ are distributed according to a posterior

f(x)~𝒩(μ(x),Σ(x)).

Therefore, the significance for an expressional change between two conditions k and l can be quantified through the Mahalanobis distance (Supp. Note 2):

$$D(a,b)=\sqrt{{\left(\mu_{a}-\mu_{b}\right)}^{T}{\left(\Sigma_{a}+\Sigma_{b}\right)}^{-1}\left(\mu_{a}-\mu_{b}\right)}$$

Notably, this adequately accounts for redundancy, as adding a nearly identical cell state contribute nearly nothing to the value of this statistic, while more distinct cell-states have a larger contribution. We leverage this to our advantage. By producing a fixed set of landmarks, using k-means clustering, we limit the evaluation of the gene-expression function and the size of the covariance matrix to a fixed size. By using a fixed number of steps and k++ initialization, both the clustering and the GP only grow linearly in run-time with the number of cells. Notably, k-means grows with O(t×k×d×n) where t is the number of iterations, k the number of landmarks, d the dimensionality of the space, and n the number of cells.

### Data analysis

Detailed descriptions of data generation and analysis described in the manuscript will be included in a subsequent version of the manuscript.

## Supplementary Material

Supplement 1

Supplement 2

## Data Availability

Raw single-cell data for the murine aging hematopoiesis will be deposited to GEO. Processed anndata object with annotations is available to download from Zenodo^36^.
