# Supplementary material for: Comparing phenotypic manifolds with Kompot: Detecting differential abundance and gene expression at single-cell resolution
Source: bioRxiv. 2025 Jun 7:2025.06.03.657769. Preprint. [Version 2] doi: 10.1101/2025.06.03.657769 (PMC12157388; doi:10.1101/2025.06.03.657769)
Supplement: Supplement 1 [file media-1.pdf]

Supplementary Note 1

Gaussian Processes and Posterior Uncertainty

In this chapter, we discuss the different sources of uncertainty for the functions we derive through a Gaussian Process (GP). We use GPs to infer cell-state density functions through *Mellon* and to compute feature-expression functions, to predict gene expression or other features from arbitrary points in the cell-state space. In both cases, we employ Bayesian inference to compute a posterior distribution of functions that quantifies our uncertainty about the *true* cell-state density or gene-expression function. Different sources of uncertainty are considered, including those arising from latent representations, measurement noise, and approximation techniques.

1.1 Theoretical Framework

Gaussian Processes (GPs) define a distribution over functions, characterized by a mean function $m(x)$ and a covariance function $k(x, x')$. For a set of observed data points $\mathcal{D} = \{(x_i, y_i)\}_{i=1}^N$, the GP posterior distribution is given by:

$$f(x) \mid \mathcal{D} \sim \mathcal{GP}\left(m_{\text{post}}(x),\, k_{\text{post}}(x, x')\right).$$

The posterior mean and covariance functions are computed as:

$$m_{\text{post}}(x) = m(x) + K_{x,X}\, K_{X,X}^{-1}\, (Y - m(X))\,, \tag{1.1}$$

$$k_{\text{post}}(x, x') = k(x, x') - K_{x,X}\, K_{X,X}^{-1}\, K_{X,x'}. \tag{1.2}$$

- $K_{X,X}$ is the covariance matrix for the observed inputs X.
- $K_{x,X}$ is the covariance vector between a test point x and the observed inputs.
- Y is the vector of observed outputs.

The covariance matrices are computed using a kernel function $K_{x,x'} = k(x, x')$ that introduces a prior statistical connection between function values based on the similarity of cell states. The details of this choice are discussed in Section 1.1.1.

1.1.1 Covariance

The prior covariance kernel encodes our assumptions about the smoothness and similarity of function values at different input locations. In our framework, the kernel $k(x, x')$ defines a statistical relationship between the function values $f(x)$ and $f(x')$ according to the distance or similarity between the corresponding cell states x and x'. This prior correlation structure is critical for capturing the underlying biological relationships in cell-state space.

While our implementation permits the free choice of covariance functions, we, by default, employ a Matérn $\frac{5}{2}$ kernel. This kernel is widely favored over alternatives such as the squared exponential or Gaussian kernel because it allows for a controlled level of roughness in the modeled functions, reflecting the moderate smoothness observed in many biological systems. The Matérn $\frac{5}{2}$ kernel strikes a good balance between flexibility and smoothness, and its well-defined derivative structure makes it particularly useful to applications that require differentiability.

A further advantage of our approach is the incorporation of a heuristic for selecting the length scale parameter of the Matérn $\frac{5}{2}$ covariance function. In the *Mellon* manuscript [2], we demonstrated that the results are remarkably stable with respect to variations in the length scale. The heuristic consistently chooses length scales that are close to the optimal values, thereby preserving the quality of the inferences. Importantly, this heuristic is the primary factor that accelerates computations, making the approach feasible for datasets comprising millions of cells, since exhaustive hyperparameter tuning would be computationally prohibitive.

1.1.2 The Sparse GP

Sparse Gaussian Processes (SGPs) address the computational limitations of standard GPs when dealing with large datasets. In SGPs, the full set of observations $\mathcal{D}$ is approximated using a smaller set of M inducing

points $Z = \{z_j\}_{j=1}^{M}$, where $M \ll N$. The key approximation is to assume that the function values $f(X)$ are conditionally independent given $f(Z)$. The joint distribution over $f(X)$ and $f(Z)$ is given by:

$$\begin{bmatrix} f(Z) \\ f(X) \end{bmatrix} \sim \mathcal{N}\left(\begin{bmatrix} m(Z) \\ m(X) \end{bmatrix}, \begin{bmatrix} K_{Z,Z} & K_{Z,X} \\ K_{X,Z} & K_{X,X} \end{bmatrix} \right).$$

From this joint distribution, the conditional distribution of $f(X)$ given $f(Z)$ is:

$$f(X) \mid f(Z) \sim \mathcal{N}\left(\mu_{X|Z}, \Sigma_{X|Z}\right),$$

where:

$$\mu_{X|Z} = m(X) + K_{X,Z}\, K_{Z,Z}^{-1}\, (f(Z) - m(Z)), \tag{1.3}$$

$$\Sigma_{X|Z} = K_{X,X} - K_{X,Z}\, K_{Z,Z}^{-1}\, K_{Z,X}. \tag{1.4}$$

Using this result, the posterior mean and covariance functions of the sparse GP are approximated as:

$$m_{\text{post}}(x) = m(x) + K_{x,Z}\, K_{Z,Z}^{-1}\, K_{Z,X}\, K_{X,X}^{-1}\, (Y - m(X)), \tag{1.5}$$

$$k_{\text{post}}(x, x') = k(x, x') - K_{x,Z}\, K_{Z,Z}^{-1}\, K_{Z,x'} + K_{x,Z}\, K_{Z,Z}^{-1}\, K_{Z,X}\, K_{X,X}^{-1}\, K_{X,Z}\, K_{Z,Z}^{-1}\, K_{Z,x'}. \tag{1.6}$$

The use of $K_{Z,Z}$, the covariance matrix over inducing points, significantly reduces the computational cost of inversion from $\mathcal{O}(N^3)$ to $\mathcal{O}(NM^2)$.

1.1.3 Latent Representation and Cholesky Decomposition

When inferring the cell-state density function with *Mellon*, we cannot directly condition the posterior on preexisting data due to the absence of explicit observations. Instead, we impose a Gaussian prior on a latent representation $z \in \mathbb{R}^M$ and link the function values to a nearest-neighbor distribution. As detailed in the *Mellon* manuscript [2], this formulation allows us to define the prior in terms of both the latent space and the function's smoothness properties:

$$z \sim \mathcal{N}(0, \mathbb{I}), \tag{1.7}$$

$$f(X) = L_X\, z + m(X), \tag{1.8}$$

$$L_X L_X^T = K_{X,Z}\, K_{Z,Z}^{-1}\, K_{Z,X} \approx K_{X,X}, \tag{1.9}$$

$$f(X) \sim \mathcal{N}\left(m(X), K_{X,X}\right). \tag{1.10}$$

Thus, instead of conditioning directly on existing data Y, we link the resulting function values to our data stochastically via $f(X) \sim g(Y)$ and perform Bayesian inference on z. The approximation $L_X L_X^T \approx K_{X,X}$ is achieved by leveraging the inducing points Z to efficiently represent the covariance structure, reducing the computational complexity associated with directly inverting $K_{X,X}$ and instead applying the Cholesky decomposition on the low-dimensional $K_{Z,Z}$. Expressing $f(X)$ in terms of z and L_X enables efficient posterior inference while respecting the underlying structure of the cell-state space. However, it also introduces an additional source of uncertainty that must be propagated from the posterior distribution of z.

1.2 Sources of Uncertainty

1.2.1 Latent Representations

For the cell-state density inference, we use a latent representation of the inferred function as explained in 1.1.3. We employ Automatic Differential Variational Inference (ADVI) [1], which produces a parameterized posterior distribution for the latent representation:

$$z \sim \mathcal{N}\left(\mu_{\text{post}}, \operatorname{diag}(\sigma^2_{\text{post}})\right). \tag{1.11}$$

This propagates into a posterior uncertainty of the mean function from (1.8):

$$m_{\text{post}}(x) \sim \mathcal{N}\left(L_x\, z + m(x), L_x \operatorname{diag}(\sigma^2_{\text{post}})\right). \tag{1.12}$$

Note that this is the posterior for the mean function at any cell state x. The posterior distribution for the actual function value includes additional terms (s. 1.3.1).

1.2.2 Measurement Noise

Feature-expression functions are conditioned on observed feature values y_i, which are typically corrupted by Gaussian noise $\epsilon \sim \mathcal{N}(0, \sigma^2)$. The noisy observations are modeled as:

$$y_i = f(x_i) + \epsilon,$$

where $f(x_i)$ is the true underlying function. The covariance function incorporates this noise term by modifying $K_{X,X}$ as:

$$K_{X,X} \to K_{X,X} + \sigma^2 I.$$

1.2.3 Sparse GP Approximation

To handle large datasets, we employ sparse GPs using inducing points $Z = \{z_j\}_{j=1}^{M}$, where $M \ll N$. The posterior distribution is defined in Section 1.1.2 with equation (1.6) for $k_{\text{post}}(x, x')$ featuring $K_{X,X}$. Note that in the case of cell-state density inference, we do not add noise to $K_{X,X}$ since we condition on the posterior mean function $f(X)$.

1.3 Applications

1.3.1 Cell-State Density Functions

For cell-state density inference, we combine the uncertainties of the posterior mean function m_{post} with the uncertainty of the function value given by k_{post}. This includes the variance of the function values at any new cell state x_i as well as the covariance between any pair of states in $\xi = \{x_i\}_{i=1}^{m}$:

$$f(\xi) \sim \mathcal{N}\left(L_\xi\, z + m(\xi),\, L_\xi\ \text{diag}(\sigma^2_{\text{post}}) + k_{\text{post}}(\xi)\right). \tag{1.13}$$

Conceptually, this is the result of stacking two inferences. First, we infer the latent representation of the function (subject to the posterior uncertainty in (1.12)). Then, we compute the function values at new cell states x_i, applying the additional uncertainty of the GP through k_{post}. Hence, $f(\xi)$ can be written as the sum of two random variables:

$$f(\xi) = a(\xi) + b(\xi), \tag{1.14}$$

$$a(\xi) \sim \mathcal{N}\left(0,\, k_{\text{post}}(\xi)\right), \tag{1.15}$$

$$b(\xi) \sim \mathcal{N}\left(L_\xi\, z + m(\xi),\, L_\xi\ \text{diag}(\sigma^2_{\text{post}})\right). \tag{1.16}$$

Here, $a(\xi)$ represents the GP's posterior uncertainty about deviations from zero, and the distribution of $b(\xi) = m_{\text{post}}(\xi)$ represents the uncertainty in the inferred mean function.

1.3.2 Feature-Expression Prediction

In feature prediction, we do not employ a latent representation of the function. However, we must account for measurement noise by modifying $K_{X,X}$ as described in Section 1.2.2. The posterior distribution for function values at cell states $\xi = \{x_i\}_{i=1}^{m}$ is:

$$f(\xi) \sim \mathcal{N}\left(m_{\text{post}}(\xi),\, k_{\text{post}}(\xi)\right). \tag{1.17}$$

Supplementary Note 2

Mahalanobis Distance

We compute functions over the cell-state space using the Gaussian Process framework discussed in previous sections. These functions can represent various biological quantities, such as cell-state density, gene expression, or other phenotypic features that vary across different cellular states. When single-cell datasets from multiple experimental conditions are available, and certain cell states are well aligned across conditions, separate GP functions can be inferred for each condition, conditioned on the respective data. This allows us to perform a quantitative comparison of any feature between conditions.

A key advantage of the GP framework is its ability to compute the posterior mean function for any feature within a given condition at any cell state x:

$$m_{\text{post}}(x).$$

This function provides our best estimate of the feature value at x, even if no direct observation is available. The associated uncertainty is captured by the posterior covariance, as discussed in Section 1.2.

2.1 Definition

The Mahalanobis distance quantifies the deviation of a point from a multivariate normal distribution while accounting for variances and covariances among dimensions. Given a point x and a normal distribution with mean 0 and covariance matrix Σ, the Mahalanobis distance is defined as:

$$D(x) = \sqrt{x^T \Sigma^{-1} x}. \tag{2.1}$$

For our analysis, we compare two experimental conditions, a and b, where the functions f_a and f_b are inferred as independent normal distributions. The covariance matrices Σ_a and Σ_b quantify both our uncertainty about the true function values and the statistical interdependencies emerging from our inference process:

$$f_a \sim \mathcal{N}\left(\mu_a, \Sigma_a\right), \tag{2.2}$$

$$f_b \sim \mathcal{N}\left(\mu_b, \Sigma_b\right). \tag{2.3}$$

Thus, the difference between the two functions is also normally distributed:

$$f_a - f_b \sim \mathcal{N}\left(\mu_a - \mu_b, \Sigma_a + \Sigma_b\right). \tag{2.4}$$

We seek to quantify the likelihood that the true difference is effectively zero. To do so, we compute the Mahalanobis distance of this difference from zero:

$$D(a, b) = \sqrt{\left(\mu_a - \mu_b\right)^T \left(\Sigma_a + \Sigma_b\right)^{-1} \left(\mu_a - \mu_b\right)}. \tag{2.5}$$

This measure quantifies the significance of the difference while accounting for uncertainty in both conditions.

2.2 Interpretation

When (2.5) is computed for a single dimension—comparing function values at a single cell state—it reduces to a standard z-score. However, when additional cell states are included, the computation generalizes to a multivariate setting:

- If the included cell states are sufficiently different, their covariances become negligible, and the Mahalanobis distance behaves like a Euclidean norm over independent z-scores.
- If cell states are highly similar (i.e., strongly correlated), their individual contributions are weighted down, preventing redundant information from dominating the measure.

Thus, the Mahalanobis distance can be viewed as a multidimensional extension of a z-score, effectively quantifying the significance of function differences across conditions while accounting for the inherent covariance in our inferences, for example, the smoothing effect introduced by gene-expression imputation.

2.3 Implementation

A central challenge in computing the Mahalanobis distance is the inversion of the covariance matrix Σ. Direct inversion can be numerically unstable, especially when Σ is nearly singular. To address this, we employ Cholesky decomposition:

$$\Sigma' = \Sigma + \epsilon\,\mathbb{I}, \tag{2.6}$$

where ϵ is a small regularization term ensuring numerical stability. The decomposition is then performed:

$$\Sigma' = L\,L^T, \tag{2.7}$$

with L a lower triangular matrix. Instead of computing Σ'^{-1} explicitly, we solve for y via forward and backward substitution:

$$L\,y = x, \tag{2.8}$$

$$D(x) = \sqrt{y^T\,y}. \tag{2.9}$$

This method avoids direct matrix inversion, improving computational efficiency and stability.

2.4 Applications

The application of the Mahalanobis distance varies depending on the type of function being compared. When comparing cell-state density functions, the covariance matrices incorporate the uncertainty of the latent function representations (denoted as σ^2_{post}; see Section 1.3.1). In contrast, when comparing other feature functions, such as gene expression, which are directly conditioned on noisy data, the relevant covariance structure is described in Section 1.3.2. This distinction ensures that the uncertainty inherent in each type of function is properly accounted for in the comparison process.

Bibliography

[1] Warren Morningstar et al. “Automatic Differentiation Variational Inference with Mixtures”. In: *Proceedings of The 24th International Conference on Artificial Intelligence and Statistics*. Ed. by Arindam Banerjee and Kenji Fukumizu. Vol. 130. Proceedings of Machine Learning Research. PMLR, 2021, pp. 3250–3258. URL: https://proceedings.mlr.press/v130/morningstar21b.html.

[2] Dominik J. Otto et al. “Quantifying Cell-State Densities in Single-Cell Phenotypic Landscapes using Mellon”. In: *Nature Methods* (June 2024). DOI: 10.1038/s41592-024-02302-w. URL: https://www.nature.com/articles/s41592-024-02302-w.
