# Supplementary material for: Comparing phenotypic manifolds with Kompot: Detecting differential abundance and gene expression at single-cell resolution
Source: bioRxiv. 2025 Jun 7:2025.06.03.657769. Preprint. [Version 2] doi: 10.1101/2025.06.03.657769 (PMC12157388; doi:10.1101/2025.06.03.657769)
Supplement: Supplement 2 [file media-2.pdf]

Table of Contents

Supplementary Figure 1: Kompot differential expression 2
Supplementary Figure 2: Kompot differential abundance 3
Supplementary Figure 3: CITE-seq dataset of murine aging hematopoiesis. 4
Supplementary Figure 4: CITE-seq dataset of murine aging hematopoiesis. 5
Supplementary Figure 5: Age-specific densities and uncertainties 6
Supplementary Figure 6: Kompot differential abundance analysis in aging hematopoiesis. 7
Supplementary Figure 7: Consistency of Kompot differential expression results with hematopoietic aging atlas 8
Supplementary Figure 8: Kompot differential expression analysis in aging hematopoiesis. 9

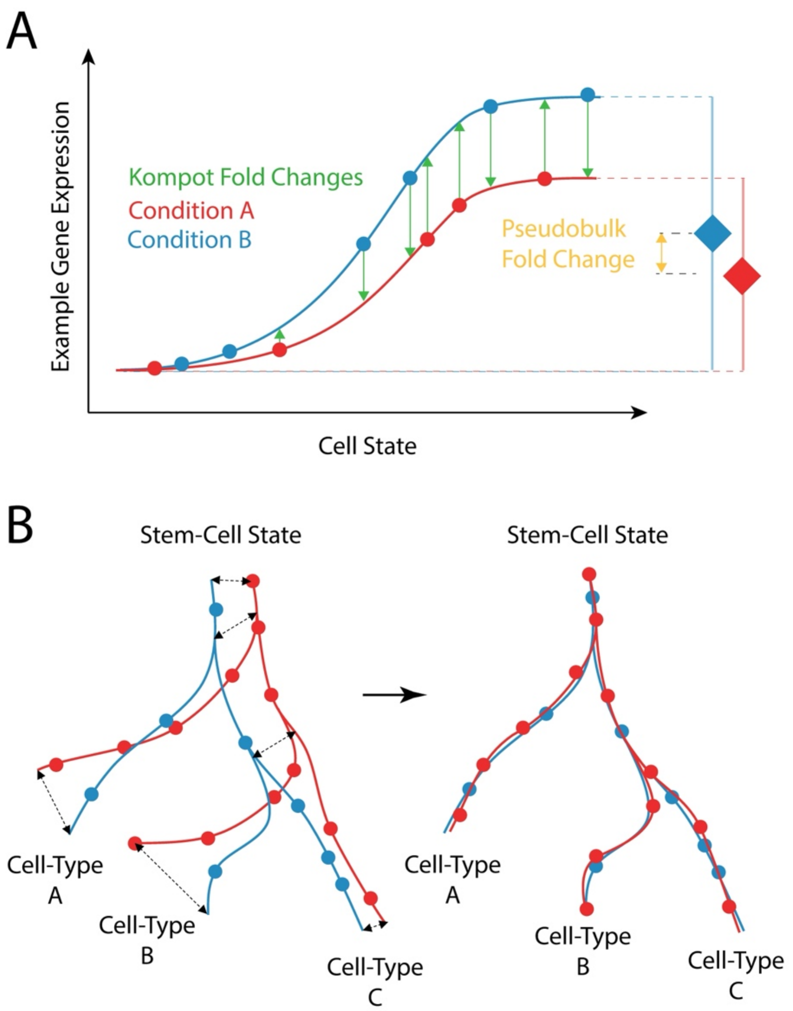

Supplementary Figure 1: Kompot differential expression

(A) Point-wise fold changes used in Kompot on the left, and diminished fold change with large confidence intervals of aggregated pseudobulk in comparison on the right.

(B) Batch correction aligns comparable cell-states in cell-state space.

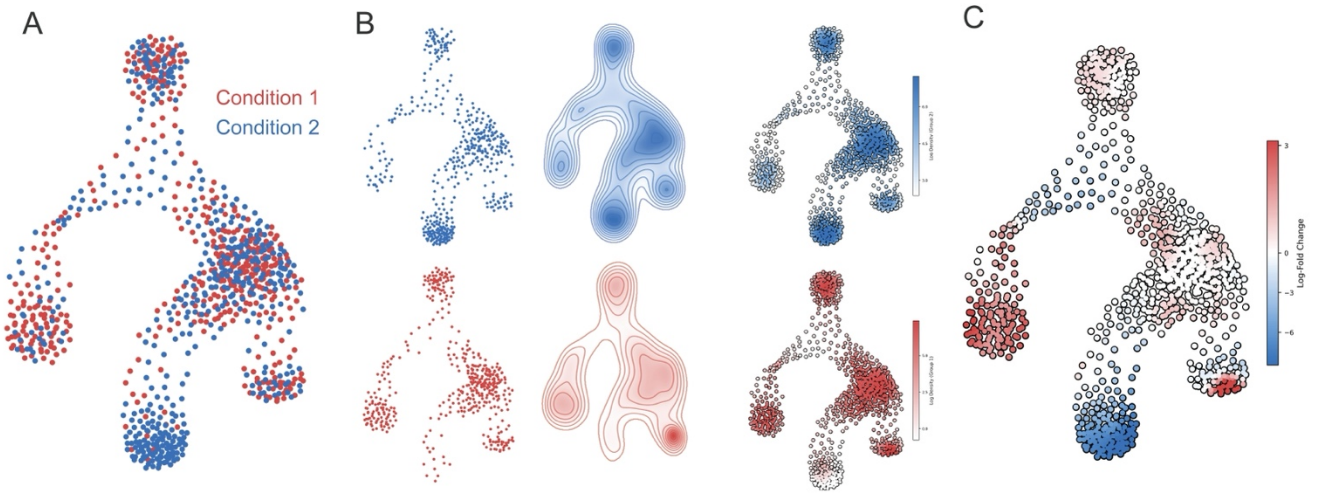

Supplementary Figure 2: Kompot differential abundance

(A) Same as 1A: Diffusion map representation of co-embedded cell-states from two conditions.

(B) Left: Subset of cells from each condition. Center: Continuous density function for each condition. Right: Condition-specific density evaluated for all cell states across conditions using the two separate density functions.

(C) Abundance log fold change computed as the ratio of the two condition specific densities at single-cell resolution.

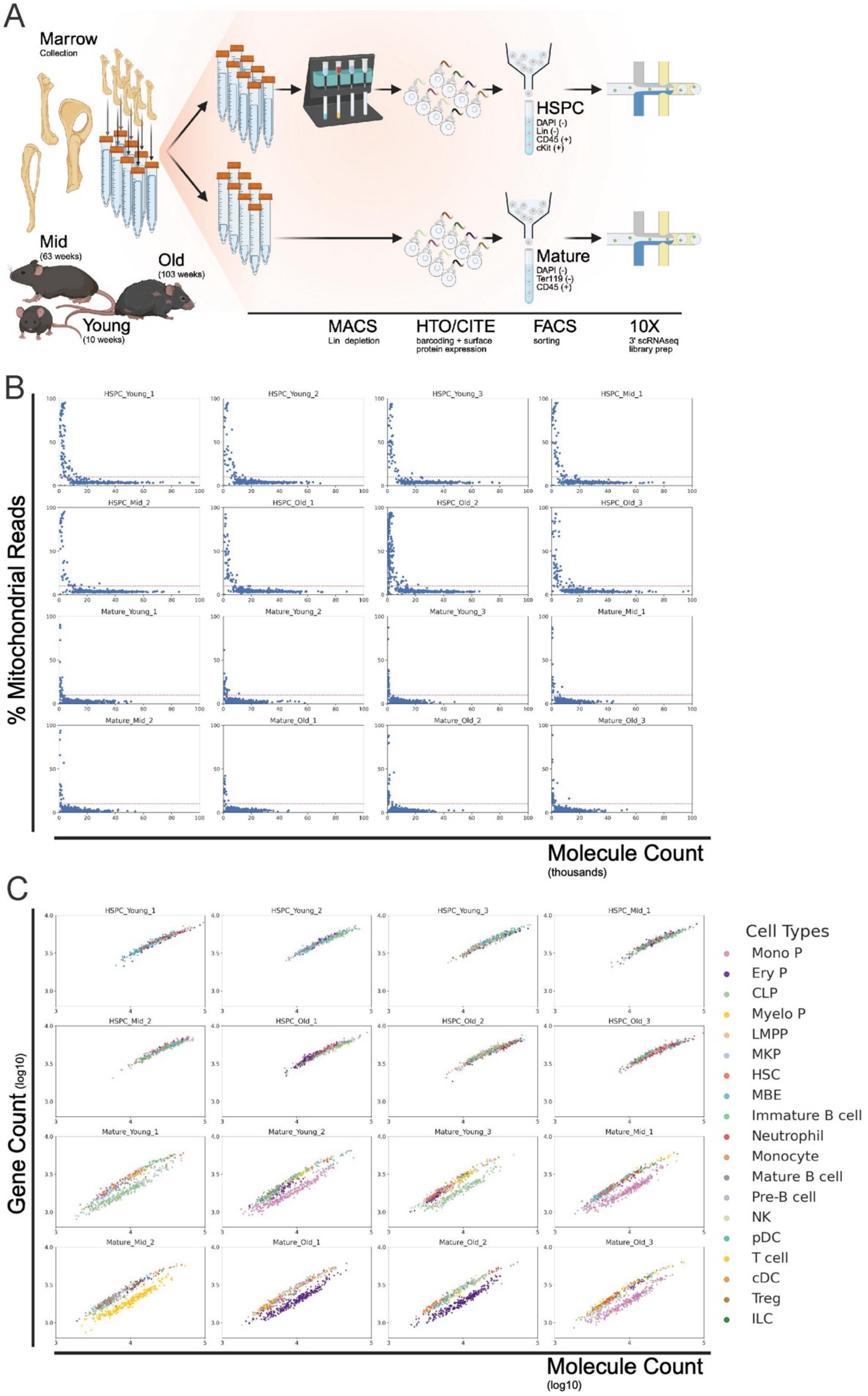

Supplementary Figure 3: CITE-seq dataset of murine aging hematopoiesis.

(A) Schematic illustrating the generation of our murine aging hematopoiesis CITE-seq dataset

(B) Plots comparing molecule counts (x-axis) and fraction of mitochondrial molecules (y-axis) for each sample. Cells with greater than 10% mitochondrial molecules were excluded from downstream analysis.

(C) Plots comparing molecule counts and gene counts for each sample. Cells are colored using annotations in Fig. 2A

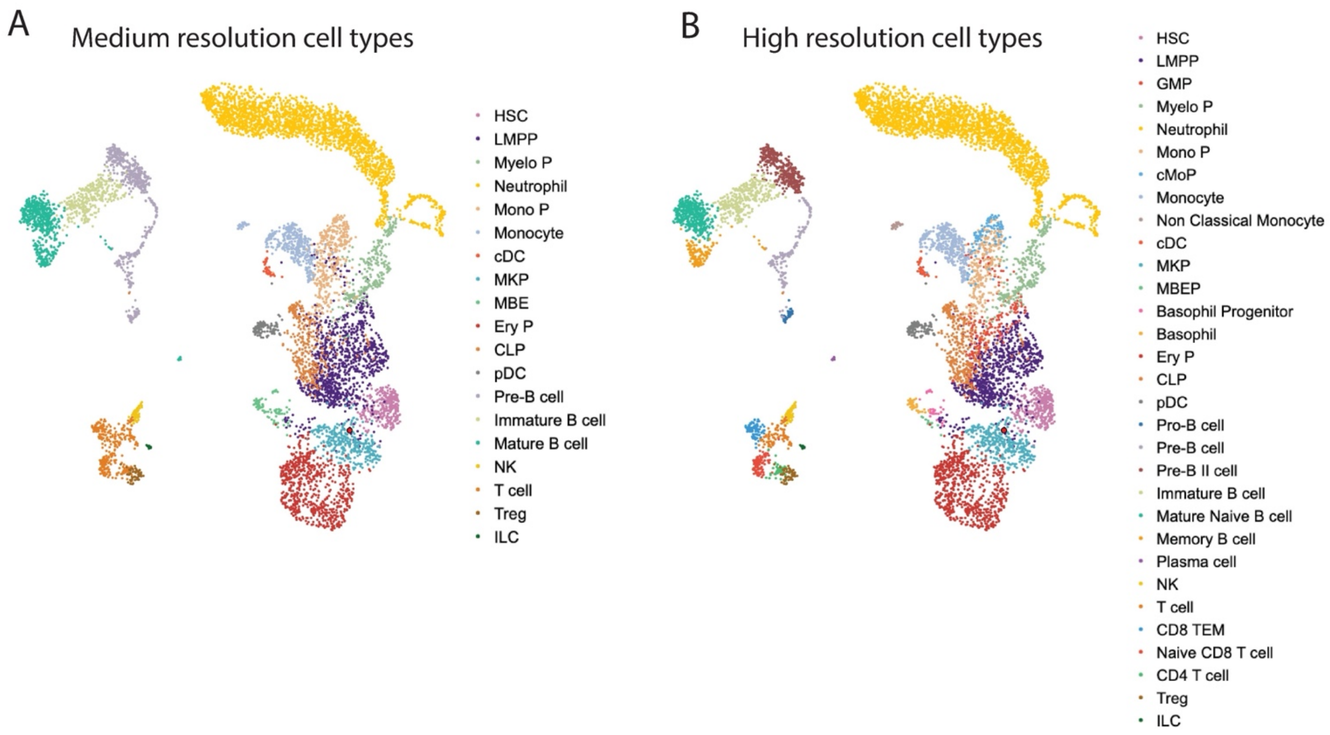

Supplementary Figure 4: CITE-seq dataset of murine aging hematopoiesis.

(A) UMAP of murine aging hematopoiesis dataset colored by medium resolution cell types.

(B) Same as (A), colored by high resolution cell types.

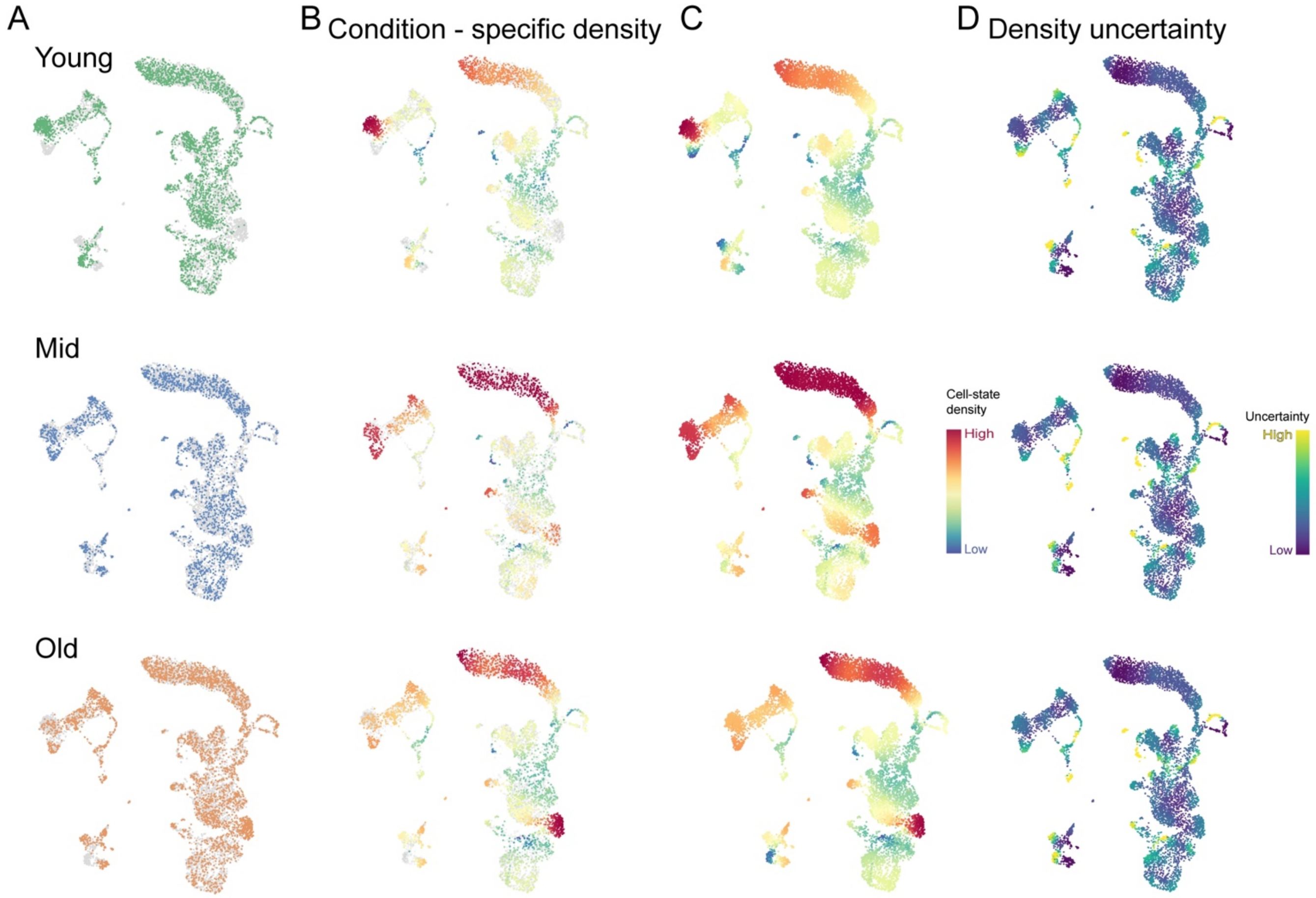

Supplementary Figure 5: Age-specific densities and uncertainties

(A) UMAPs highlighting cells from different age (Same as Fig. 2B).

(B) Age-specific density computed using the subset of cells in (A).

(C) Age-specific density evaluated all cells using the continuous density function.

(D) Uncertainties associated with density estimates in (C).

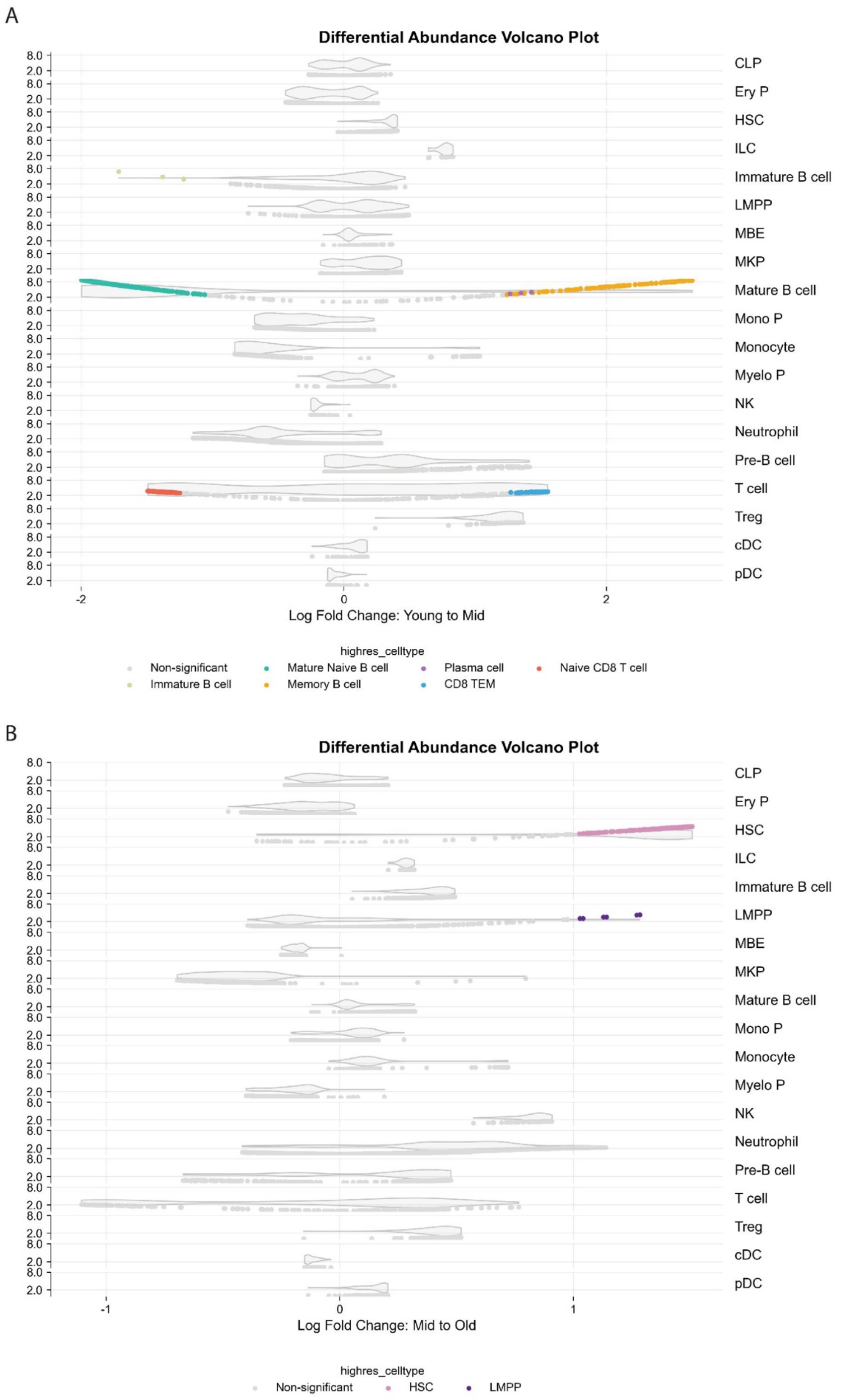

Supplementary Figure 6: Kompot differential abundance analysis in aging hematopoiesis.

Same as Fig. 2F, for Young to Mid and Mid to Old comparisons.

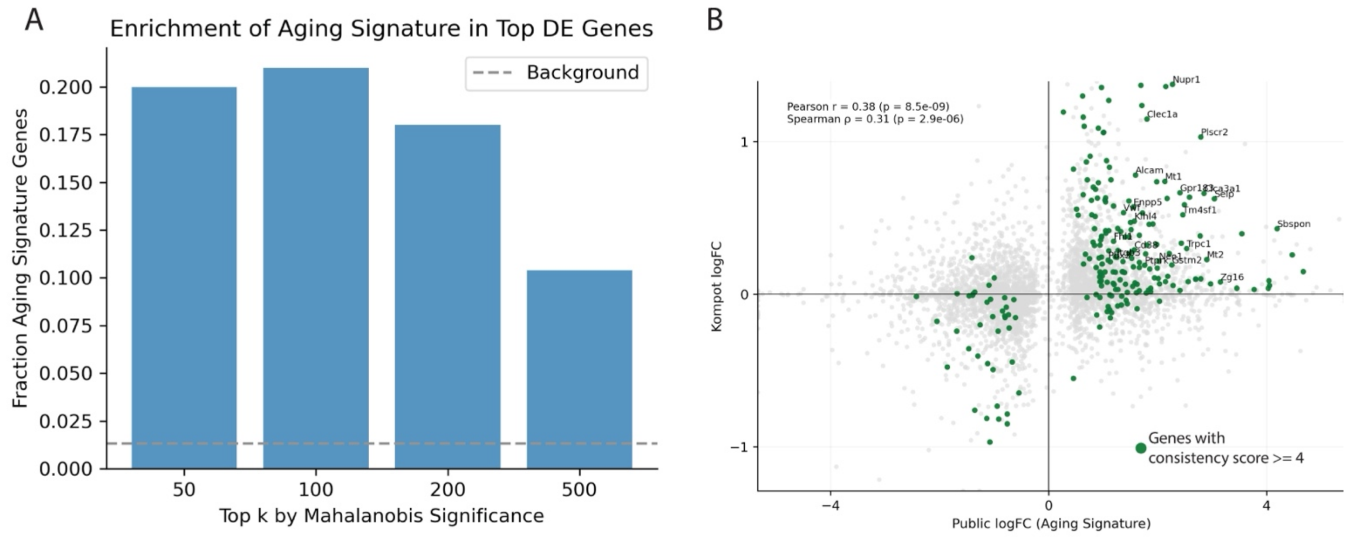

Supplementary Figure 7: Consistency of Kompot differential expression results with hematopoietic aging atlas

(A) Enrichment of aging-associated genes among Kompot's top differentially expressed genes (DEGs). Bars show the fraction of genes within the top k ranked by Mahalanobis significance that are part of a published hematopoietic aging signature, defined as genes with a reported consistency score ≥ 4. The dashed horizontal line indicates the background frequency of such genes across all genes tested.

(B) Comparison of average log-fold changes (logFC) per gene between Kompot (Mid to Old transition; y-axis) and the published aging signature (x-axis). Genes with consistency scores ≥ 4 are highlighted in green; those with scores ≥ 8 are additionally labeled. Pearson and Spearman correlation coefficients between the two datasets are shown.

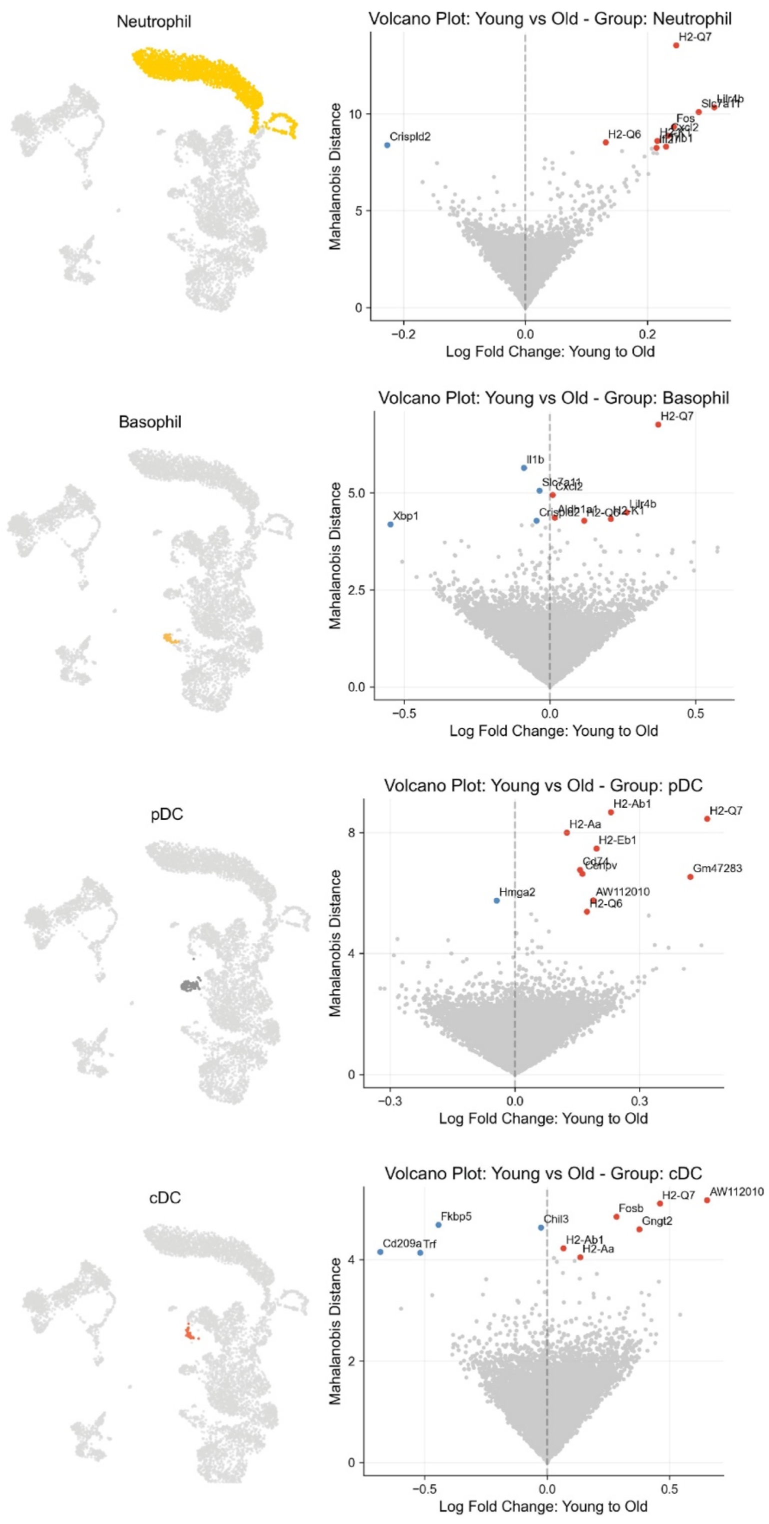

Supplementary Figure 8: Kompot differential expression analysis in aging hematopoiesis.

Same as Fig. 3F, for neutrophils, basophils, pDCs and cDCs.
